# Variability in recurrence rates with acute treatments for migraine: why recurrence is not an appropriate outcome measure

**DOI:** 10.1186/s10194-022-01519-4

**Published:** 2022-11-22

**Authors:** Stewart J. Tepper, Jessica Ailani, Sutapa Ray, Joe Hirman, Stephen B. Shrewsbury, Sheena K. Aurora

**Affiliations:** 1 Geisel School of Medicine at Dartmouth, Hanover, NH USA; 2 MedStar Georgetown University Hospital, Washington, DC USA; 3 Impel Pharmaceuticals, Seattle, WA USA; 4 Pacific Northwest Statistical Consulting, Inc, Woodinville, WA USA

**Keywords:** Dihydroergotamine, Recurrence, Sustained pain freedom, Sustained pain relief, Rescue medication

## Abstract

**Background:**

Headache recurrence is a common feature of acute therapies, whether approved or still in development, and continues to be a significant problem for both the patient and the clinician. Further complicating this issue is lack of standardization in definitions of recurrence used in clinical trials, as well as disparity in patient characteristics, rendering a comparison of different acute medications challenging. Recurrence has serious clinical implications, which can include an increased risk for new-onset chronic migraine and/or development of medication overuse headache. The aim of this review is to illustrate variability of recurrence rates depending on prevailing definitions in the literature for widely used acute treatments for migraine and to emphasize sustained response as a clinically relevant endpoint for measuring prolonged efficacy.

**Body:**

A literature search of PubMed for articles of approved acute therapies for migraine that reported recurrence rates was performed. Study drugs of interest included select triptans, gepants, lasmiditan, and dihydroergotamine mesylate. An unpublished post hoc analysis of an investigational dihydroergotamine mesylate product that evaluated recurrence rates using several different definitions of recurrence common in the literature is also included. Depending on the criteria established by the clinical trial and the definition of recurrence used, rates of recurrence vary considerably across different acute therapies for migraine, making it difficult to compare results of different trials to assess the sustained (i.e., over a single attack) and the prolonged (i.e., over multiple attacks) efficacy of a particular study medication.

**Conclusion:**

A standardized definition of recurrence is necessary to help physicians evaluate recurrence rates of different abortive agents for migraine. Sustained pain relief or freedom may be more comprehensive efficacy outcome measures than recurrence. Future efficacy studies should be encouraged to use the recommended definition of sustained pain freedom set by the International Headache Society.

## Introduction

Treatment efficacy in migraine clinical trials can be assessed using a range of possible outcome measures, from symptomatic relief at specific time points post-treatment to persistence of treatment response [1]. The International Headache Society (IHS) Clinical Trials Standing Committee recommends using 2-h pain freedom as the primary efficacy endpoint, with sustained pain freedom as an important secondary endpoint. The IHS defines sustained pain freedom as the percentage of patients who are “pain free at 2 h with no use of rescue medication or relapse within 24 or 48 h of the initial treatment” [2]. Indeed, sustained pain freedom may be the ideal treatment response, as it includes headache resolution, lack of recurrence, and lack of rescue medication usage [3]. Further, in trials in which different interventions have been compared, sustained pain freedom was recommended over recurrence as an endpoint [2, 3]. The IHS classifies recurrence of migraine as “the occurrence of headache of any severity within 48 h of the administration of an investigational treatment among subjects who were pain free 2 h after the investigational treatment was administered.” If the treatment has a short half-life, the occurrence of headache of any severity within 24 h of initial pain freedom can be used as a definition of recurrence [2].

Complete relief of head pain and no recurrence are important attributes of acute therapy for migraine identified from surveys of individuals with migraine [4, 5]. Lack of headache recurrence following initial migraine therapy is an important predictor of improved health-related quality of life [6]. Recurrence of headache pain can lead to repeat dosing with acute medications for migraine, which can prove problematic, as some acute treatments are limited in the number of doses that can be taken within a certain period, and repeat dosing may result in medication overuse and medication overuse headache, as well as other drug-induced health issues over time [7–10]. Importantly, there is a lack of published data available on the efficacy and/or safety of repeat dosing. Further, repeat dosing for a specific attack has cost implications, which, for some patients, may lead to anxiety about running out of a limited supply of acute medication [10, 11]. Lastly, suboptimal acute treatment can be associated with an increased likelihood of transformation from episodic migraine to chronic migraine [12].

Despite advances in migraine care, recurrence of migraine remains a major problem for patients and providers alike, which requires careful consideration when evaluating treatment efficacy in clinical trials [2, 13]. Several factors may be associated with headache recurrence, including patient age, less-severe attacks (moderate vs severe migraine) or treatment at mild pain, duration of attack, slow drug-receptor dissociation, and characteristics of the patient and the particular attack [13]. Importantly, various definitions of recurrence have been used in the literature of clinical trials. Recurrence is typically defined as the return of headache pain after initial pain relief, but even pain relief itself and the time point at which efficacy should be measured have different definitions. The initial definition of pain relief, also known as headache relief or headache response, was reduction in pain from moderate or severe (2–3 on a scale of 0–3) to mild or no pain (1 or 0), measured at 2 h post-treatment [9, 14, 15]. In some studies, however, in particular those involving the use of noninvasive neuromodulation devices, pain relief was defined as either severe or moderate pain moving to mild or none, or mild pain at treatment moving to no pain at a prespecified time after treatment [16]. Some trials measure the return of headache after pain relief, whereas others do so following pain freedom, that is, 2–3 to 0, or mild, moderate, or severe pain to no pain, depending on the study. The return of headache pain can also be defined as the return of moderate to severe pain, any headache pain, or even headache pain that is perceived as being clinically meaningful by the patient. The return of headache pain can be defined as the return of pain up to 24 h following pain relief or pain freedom at 2 h or at 4 h. Occasionally, recurrence is determined in the window of time from pain relief or pain freedom to 48 h post-pain relief or freedom. In some cases, patients using rescue medication are also considered to have experienced a recurrence, as the need for rescue medication implies that pain is once again present in that patient. The need to take additional medication may represent a failure of the initial dose, however, and not a true return of headache. Because multiple definitions of recurrence are in use, with most clinical trials identifying a single definition, it is difficult to compare recurrence rates across clinical trials to evaluate the sustained or prolonged efficacy of various migraine therapies. In this narrative review, we examine the different definitions of recurrence used in clinical trials evaluating the efficacy of acute treatments for migraine and demonstrate the significance of using a standard definition; we also emphasize the importance of sustained response as a more clinically relevant endpoint for measuring sustained or prolonged efficacy.

## Methods

A literature search of PubMed for clinical trials of approved acute therapies for migraine that reported recurrence rates was performed. No restriction was placed on the publication year. Study drugs of interest included ubrogepant, rimegepant, lasmiditan, INP104, and triptans. For the purpose of this review, in order to simplify issues with medication evaluation, we did not include noninvasive neuromodulation devices used to acutely treat migraine. Studies of primary focus were manually identified and were included if they were clinical trials (i.e., randomized, open-label, active- or placebo-controlled), provided any definition of recurrence, and reported recurrence rates.

The focus of this review was on pain rather than on the other, often debilitating, symptoms of migraine. For the agent rimegepant, only studies that evaluated the orally disintegrating tablets (ODTs) were included, because this formulation has been approved by the US Food and Drug Administration (FDA) [17]. The literature search for triptans and recurrence provided us with many comprehensive reviews on this topic. To focus this review and not repeat published work, we decided to limit this work to two key studies that assessed recurrence with oral sumatriptan use, since sumatriptan is the most commonly prescribed acute treatment for migraine, along with studies that evaluated frovatriptan, as this is a triptan with a longer half-life. We also included an unpublished post hoc analysis of MAP0004 (LEVADEX/SEMPRANA™, MAP Pharmaceuticals Inc., Irvine, CA), an orally inhaled formulation of dihydroergotamine (DHE) mesylate that was evaluated in clinical trials [18]. Some of this analysis was presented in abstract form [19]. MAP0004 is not approved by the FDA, and the development of MAP0004 was halted because of manufacturing issues [18, 20]. Data from this analysis will be reviewed nonetheless, because of the importance of demonstrating the variability in recurrence rates. The MAP0004 data are unpublished and were provided by correspondence with one of the authors (SJT). Two authors of this manuscript had access to the MAP0004 data and were closely involved in the analyses (SJT, SKA).

## Recurrence of migraine in clinical trials assessing acute treatments for migraine

### Sumatriptan and frovatriptan

The recognition of recurrence as a clinical concern, which did not arise until 1989, was generated by the introduction of sumatriptan as an abortive therapy for patients with migraine [21]. Recurrence was not evaluated as part of migraine clinical trial programs before the advent of the triptan class of agents, with its documentation beginning with patient follow-up assessments that reported sustained pain responses only for a small proportion of patients and/or personal accounts from clinicians during the sumatriptan trial program [13, 21–23]. Since then, numerous studies assessing the recurrence rates of different triptan products have been conducted, and several comprehensive reviews have been published [3, 9, 13, 24]. For this review, we chose to highlight key studies that focused specifically on the evaluation of recurrence rates. The trials used different methodologies for defining recurrence and, as noted, described recurrence with oral sumatriptan, the first approved agent in the triptan class and the most commonly prescribed, and frovatriptan, a second-generation triptan with a longer plasma half-life of 26 h, compared with 2.5 h for sumatriptan [9, 13, 25, 26]. Of note, Ferrari and colleagues published several papers on the topic of recurrence, but we did not include their 1994 study on sumatriptan recurrence in our review, because that paper was an analysis of recurrence following a second dose of sumatriptan, not following the initial dose [27].

In 1995, Pini and colleagues conducted a double-blind, randomized, placebo-controlled, parallel-group, multicenter study that evaluated the incidence of recurrence in 238 patients with migraine following administration of a 100 mg dose of oral sumatriptan succinate for a single attack. Patients were instructed to take sumatriptan or placebo at the earliest sign of a migraine. Rescue medication, except for ergotamine tartrate, was permitted after 4 h post-dose.

Recurrence was defined as the reemergence of moderate or severe headache within 24 h of dosing after initial headache relief (mild or none) 4 h after treatment. With sumatriptan, 65% (92 of 142) of patients experienced headache relief at 4 h post-dose, compared with 40% (32 of 80) of those treated with placebo. In the sumatriptan group, 17.4% (16 of 92) of patients experienced recurrence from 4 to 24 h after the initial dose, compared with 12.5% (4 of 32) of those in the placebo group; these differences were not statistically significant (Table 1). This study highlighted the fact that recurrence occurred only in patients treated with sumatriptan and placebo who had a history of headache duration of > 24 h and that the recurrent headaches did not meet the IHS diagnostic criteria for migraine, suggesting that these recurrent events may not necessarily be a prolongation of the treated attacks [28]. Note that this study evaluated the return of headache following headache relief at 4 h, not at 2 h, which later became the standard time endpoint for efficacy analyses.

**Table 1:** Recurrence rates for acute treatments for migraine

**Study Drug**	**Study Type**	**Dose**	**Definition of Recurrence**	**Recurrence Population**	**Number of Patients (Denominator)**	**Recurrence Rate (%)**
**Sumatriptan ** [28]	RCT, placebo-controlled	100 mg	Reemergence of moderate or severe headache within 24 h of dosing after initial pain relief (mild or none) at 4 h	Patients who reported pain relief at 4 h	92	17.4%
		Placebo			32	12.5%
**Sumatriptan ** [14]	RCT, placebo-controlled	100 mg	Return of moderate or severe headache within 24 h of dosing after initial pain relief at 4 h (measured across 3 attacks)	Patients who reported pain relief at 4 h	1st attack: 196 2nd attack: 194 3rd attack: 179	30% 29% 28%
		50 mg			1st attack: 198 2nd attack: 194 3rd attack: 167	34% 29% 34%
		25 mg			1st attack: 167 2nd attack: 161 3rd attack: 148	34% 39% 26%
		Placebo			1st attack: 34 2nd attack: 25 3rd attack: 22	35% 48% 41%
**Frovatriptan ** [26] **(3 studies combined)**	RCT, placebo-controlled	2.5 mg	Grade 3 or 2 headache improving to grade 1 or 0 at 4 h, but subsequently returning to grade 2 or 3 within 24 h of initial dose	Patients who responded at 4 h	1454	10%-25%
		Placebo			740	24%-31%
**Lasmiditan ** [3, 29] **(SAMURAI and SPARTAN)**	RCT, placebo-controlled, pooled analysis	200 mg	Administration of a second dose if moderate or severe pain returns from 2 to 24 h	Patients who reported pain freedom at 2 h	396	7%
		100 mg			337	10%
		50 mg			169	8%
		Placebo			206	10%
		200 mg	Patients who reported none or mild pain at 2 h after the first dose and subsequently reported moderate or severe pain within 24 h	Patients who reported mild or no pain at 2 h	683	14.1%
		100 mg			698	15.3%
		50 mg			346	15.3%
		Placebo			505	17.4%
**Lasmiditan ** [30] **(GLADIATOR)**	Open-label, randomized	All doses combined	Number of attacks treated with a second dose up to 48 h after the first dose for recurrence	Patients who reported pain freedom at 2 h (ITT population)	4963	6.1%
		200 mg			2665	5.6%
		100 mg			2298	6.6%
		All doses combined	Number of attacks that had recurrence up to 48 h post-dose	Patients who reported pain freedom at 2 h (mITT population)	NA	17.1%
**Rimegepant (ODT) ** [31]	RCT, placebo-controlled	75 mg	Return of headache (some pain) within 48 h of an initial response to treatment after initial pain freedom at 2 h	Patients who reported pain freedom at 2 h	142	36.6%
		Placebo			74	50%
**Ubrogepant ** [32] **(ACHIEVE I)**	RCT, placebo-controlled	Rescue 100 mg 50 mg Placebo	Administration of a second dose of study drug or rescue medication for moderate or severe headache from 2 to 48 h after the initial dose	mITT population	448 423 456	15.2% 16.3% 28.7%
		Second Dose 50 mg/100 mg			871	38.6%
**Ubrogepant ** [33] **(ACHIEVE II)**	RCT, placebo-controlled	Rescue 50 mg 25 mg Placebo	Administration of a second dose of study drug or rescue medication for moderate or severe headache from 2 to 24 h after the initial dose	mITT population	NA NA NA	16.4% 20.5% 25.7%
		Second Dose 25 mg/50 mg Placebo			899 456	37.6% 42.8%
**MAP0004 (FREEDOM-301)**	RCT, placebo-controlled, post hoc analysis	1 mg	Return of moderate or severe pain within 24 h or 48 h of dosing (Definition A)	Patients who reported pain relief at 2 h	231	24 h: 6.5% 48 h: 10.4%
		Placebo			136	24 h: 14.7% 48 h: 17.6%
		1 mg	Return of moderate or severe pain within 24 h or 48 h of dosing OR use of rescue medications (Definition B)	Patients who reported pain relief at 2 h	231	24 h: 21.6% 48 h: 29.4%
		Placebo			136	24 h: 39.0% 48 h: 41.2%
		1 mg	Return of moderate or severe pain within 24 h or 48 h of dosing (Definition C)	mITT population	397	24 h: 3.8% 48 h: 6.0%
		Placebo			397	24 h: 5.0% 48 h: 6.0%
		1 mg	Return of moderate or severe pain within 24 h or 48 h of dosing OR use of rescue medications (Definition D)	mITT population	397	24 h: 12.6% 48 h: 17.1%
		Placebo			397	24 h: 13.4% 48 h: 14.1%
**INP104** **(STOP 301) ** [34]	Open-label	1.45 mg	Onset of a new headache prior to 24 h and 48 h post-dose	Patients who self-reported pain freedom at 2 h	126	24 h: 7.1% 48 h: 14.3%

*h* Hour, *ITT* Intent-to-treat, *mITT* Modified intent-to-treat, *NA* Not available, *ODT* Orally disintegrating tablet, *RCT* Randomized controlled trial

In 1998, Pfaffenrath and colleagues conducted a large, double-blind, parallel-group, multinational study that investigated the safety and efficacy of three doses of oral sumatriptan, 25 mg, 50 mg, and 100 mg, across three migraine attacks in 1,003 patients with migraine. Rescue medication, except for ergotamine tartrate or sumatriptan, was permitted 4 h after the initial dose. Recurrence was defined as the return of moderate or severe headache within 24 h of dosing after initial headache relief (to mild or none) at 4 h, again using the 4-h effectiveness time for initial relief. Over three attacks, 77% to 83% of patients in the sumatriptan 100 mg group, 76% to 81% in the sumatriptan 50 mg group, 65% to 70% in the sumatriptan 25 mg group, and 33% to 39% in the placebo group experienced headache relief by 4 h post-dose, with 28% to 30% of patients in the sumatriptan 100 mg group, 29% to 34% in the sumatriptan 50 mg group, 26% to 39% in the sumatriptan 25 mg group, and 35% to 48% in the placebo group experiencing recurrence from 4 to 24 h post-dose (Table 1). This study highlighted the importance of calculating recurrence from response to a single migraine attack, the last migraine attack assessed in a long-term study, or the mean rate of many attacks over time, as recurrence rates varied between migraine attacks in this study. The median time to recurrence for all three attacks was also captured, which increased with increasing sumatriptan doses for all three migraine attacks [14].

In 2002, Ryan and colleagues reported the results from three randomized, placebo-controlled, double-blind clinical studies that assessed the clinical efficacy of oral frovatriptan 2.5 mg for the acute treatment of migraine in a total of 2,676 patients. The primary endpoint was 2-h headache response in Study 1, whereas Study 2 used 2- and 4-h headache response, as well as 24-h headache recurrence, and Study 3 used 2-h headache response and 24-h headache recurrence. Headache response was defined as severe or moderate headache (grade 3/2) becoming mild or absent (grade 1/0) in all three studies. Headache recurrence at 24 h was defined as “grade 3 or 2 headache improving to grade 1 or 0, but subsequently returning to grade 2 or 3 within 24 h” of the first dose in patients who responded at 4 h. Response at 2 and 4 h ranged from 37 to 46% and 56% to 65% with frovatriptan, compared with 21% to 27% and 31% to 38% with placebo, respectively, in all three studies. The percentage of patients who were pain free at 2 and 4 h ranged from 9 to 14% and 27% to 32% with frovatriptan, compared with 2% to 3% and 9% to 14% with placebo, respectively, in all three studies. Overall, 47% to 51% of patients in the frovatriptan group, compared with 22% to 27% of those in the placebo group, reported a sustained response (i.e., proportion of the total population with a headache response at 4 h and no recurrence at 24 h). The rate of recurrence within 24 h of the first dose ranged from 10 to 25% with frovatriptan, compared with 24% to 31% with placebo in all three studies (Table 1). The mean time to recurrence was longer with frovatriptan than with placebo in all three studies [26].

Triptans are a widely used and effective acute treatment for migraine; however, the studies discussed above highlight that these agents are often associated with high and variable recurrence rates [3, 13, 14, 26, 28, 35]. The lack of uniformity with respect to how recurrence rates are defined is also evident when comparing different routes of administration of triptans. A review article that discusses the comparative efficacy of different triptans highlighted the significant variation in recurrence rates with different routes of sumatriptan administration (100 mg dose), ranging from 27 to 44% [24]. A large meta-analysis of 53 triptan trials was published in 2001 by Ferrari and colleagues. The researchers emphasized the difficulty involved in comparing recurrence rates across the triptan class, proposing a set of criteria to compare the efficacy of oral triptans by including both recurrence rates (as a proportion of responders) and sustained pain freedom (i.e., “the proportion of patients who were pain free by 2 h post-dose and who did not have a recurrence of moderate or severe headache and who did not use any rescue headache medication 2–24 h post-dose”). The authors noted that sustained pain freedom is the “ideal efficacy endpoint,” albeit a difficult one to achieve, as it requires patients to report pain freedom from their migraine attack at 2 h and for at least 24 h with a single dose of study medication [3].

Because of some negative outcomes associated with repeat dosing of acute medications (i.e., risk of medication overuse headache, cost implications), single-dose efficacy becomes important. Recurrence may be prevented or reduced with the use of an agent with a long plasma elimination half-life or slow receptor dissociation [13, 36]. A large meta-analysis of 31 placebo-controlled triptan trials conducted in 2003 by Géraud and colleagues found that the use of triptans with longer half-lives and greater potency for the 5-HT_1B_ receptor was associated with lower rates of recurrence [36]. Interestingly, Ryan and colleagues reported that the use of triptans with longer half-lives, such as frovatriptan, does not always result in lower headache recurrence, suggesting that the pharmacokinetic/pharmacodynamic relationship may be more complicated [13, 26, 37]. Further, in 2008, Tfelt-Hansen reported that the recurrence rates for sumatriptan (31%) and frovatriptan (25%) were in the same range based on data from a double-blind, randomized controlled trial that compared frovatriptan 2.5 mg and sumatriptan 100 mg with placebo [38]. Recurrence can also occur at the same time, regardless of whether a triptan has a short half-life (e.g., rizatriptan) or a longer half-life (e.g., naratriptan). In a 1999 study by Bomhof and colleagues, although recurrence at 24 h was lower with naratriptan (21%) than with rizatriptan (33%), the time of recurrence was the same at a little more than 12 h post-dose with both agents. Time to recurrence, however, was longer with both triptans than with placebo (6.9 h) [39].

### Dihydroergotamine (DHE) Mesylate (MAP0004)

DHE has been used and recommended for the treatment of migraine since 1946 [40]. DHE interacts with several receptor families and subtypes [41], which may explain how it can provide sustained efficacy for migraine symptoms even when administered late after onset of a migraine attack and even in patients who experience difficult-to-treat migraine attacks [42–46]. DHE is currently available in multiple routes of administration, including intravenous (for in-hospital use), intramuscular or subcutaneous injection, and as a nasal spray [40, 47, 48]. However, recent recurrence data for FDA-approved DHE mesylate products are limited.

As mentioned earlier, MAP0004 was an orally inhaled formulation of DHE mesylate, but it did not receive FDA approval [18, 20]. In 2009, MAP0004 was evaluated in the large, randomized, placebo-controlled, double-blind, Phase 3, FREEDOM-301 Study and was shown to be effective for the acute treatment of migraine [18]. Recognizing that difficulty exists in comparing recurrence rates across clinical trials, the investigators conducted a post hoc analysis of FREEDOM-301 to evaluate migraine recurrence and thus identify the most appropriate standardized definition of the term. Overall, 903 patients were randomized in a 1:1 ratio to either MAP0004 (1.0 mg nominal (ex-valve) dose of DHE mesylate) or placebo to treat a single qualifying migraine that occurred over a possible treatment period of 8 weeks. A qualifying migraine was a moderate or severe migraine for which the patient had not used any triptan or ergot during the previous 24 h [18]. No rescue medications were permitted during the first 2 h following study drug administration. After 2 h, non-ergot and/or non-triptan rescue medications were permitted. The recurrence rate was analyzed among patients who achieved an initial reduction in pain severity from moderate or severe intensity to mild or no pain 2 h after treatment (i.e., pain relief). Recurrence was defined as the return of moderate or severe pain within 24 or 48 h and was determined separately for each of the following 4 definitions:

(A) recurrence in patients who experienced pain relief at 2 h
(B) recurrence or use of rescue medication in patients who experienced pain relief at 2 h
(C) recurrence in the modified intent-to-treat (mITT) population, and
(D) recurrence or use of rescue medication in mITT patients

The mITT population was defined as patients who treated a qualifying migraine and for whom ≥ 1 post-treatment assessments were available. The assessments were the usual primary and secondary outcome measures, including 2-h pain status, recurrence, rescue medications, and 24-h pain status. Sustained pain relief (i.e., achieving pain relief at 2 h with no increase in pain severity or use of rescue medications for 24 h) from 2 to 24 h was reported among 44% (167 of 382) of patients treated with MAP0004, compared with 20% (76 of 387) of those treated with placebo [18]. Further, the therapeutic gain (i.e., active minus placebo response) for sustained pain relief rates at 2 to 24 h post MAP0004 nominal (ex-valve) doses of 1.0 mg and 2.0 mg has been reported to be 25% and 26%, respectively [49].

When the most commonly used definition in clinical trials (Definition A) was applied, recurrence was reported in 6.5% (15 of 231) of patients treated with MAP0004 and 14.7% (20 of 136) of those treated with placebo over 2 to 24 h after the initial dose. Recurrence varied, however, when Definitions B and C were used. Recurrence was reported in 21.6% (50 of 231) of MAP0004-treated patients vs 39.0% (53 of 136) of placebo-treated patients when Definition B was used, whereas recurrence was reported in 3.8% (15 of 397) of patients treated with MAP0004 compared with 5.0% (20 of 397) of those treated with placebo when Definition C was used. When Definition D was applied, recurrence was reported in 12.6% (50 of 397) of patients treated with MAP0004 vs 13.4% (53 of 397) of those treated with placebo. The same trend was observed when recurrence was recorded from 2 to 48 h after the initial dose. When Definition A was used, recurrence occurred in 10.4% (24 of 231) of patients treated with MAP0004 and 17.6% (24 of 136) of those who received placebo. Recurrence was reported in 29.4% (68 of 231) of MAP0004-treated patients vs 41.2% (56 of 136) of placebo-treated patients when Definition B was applied. With Definition C, recurrence was 6.0% (24 of 397) in the MAP0004 group and 6.0% (24 of 397) in the placebo group. When Definition D was used, recurrence was reported in 17.1% (68 of 397) of patients treated with MAP0004 compared with 14.1% (56 of 397) of those who received placebo (Table 1).

This post hoc analysis study provided a direct comparison of recurrence rates using different definitions of recurrence, thus highlighting how the definition can substantially change the rate of recurrence reported. Definition A (i.e., recurrence in patients who experienced pain relief at 2 h) is consistent with the most common criteria defining recurrence in the literature, in which use of rescue medication is not taken into consideration when calculating recurrence rates. For example, in the original presentation of the FREEDOM-301 study outcomes, Definition A was used, with recurrence at 24 h reported in 6.5% of patients administered MAP0004 compared with 14.7% of patients administered placebo. This definition, however, may provide a false impression, as sustained pain relief from 2 to 24 h (i.e., pain relief at 2 h with no increase in pain severity or use of rescue medications for 24 h) was achieved by 44% of patients treated with MAP0004 vs 20% of those who received placebo [18, 19]. Definition B (i.e., recurrence or use of rescue medication in patients who experienced pain relief at 2 h) paints a more complete picture of headache response because it includes the use of rescue medication. The substantially higher percentages of patients with recurrence reported for MAP0004 and placebo when Definition B was used better describes a more clinically meaningful patient response. In contrast to Definitions A and B, which are restricted to patients who experienced pain relief at 2 h in the denominator, Definitions C (i.e., recurrence in the mITT population) and D (i.e., recurrence or use of rescue medication in the mITT population) use the total mITT population in the denominator, which includes patients who did not respond to the treatment, thus artificially lowering the recurrence rate. The interpretation of data from this MAP0004 analysis is limited by several factors. It is important to note that recurrence was evaluated in a post hoc analysis, and the FREEDOM-301 study was not originally powered for this endpoint using any of the proposed definitions. These data, however, do highlight the need for future studies to clearly define *recurrence* and potentially to include patients taking rescue medications among patients who are considered to have experienced recurrence.

### Rimegepant and ubrogepant

Gepants are a class of small molecules that act as antagonists to the calcitonin gene-related peptide receptor [15, 31–33, 50]. Rimegepant (Nurtec® ODT, Biohaven Pharmaceuticals, Inc., New Haven, CT) and ubrogepant (Ubrelvy®, Allergan USA Inc., Madison, NJ) are currently the only FDA-approved gepants available for the acute treatment of migraine at the time of this writing (July 2022) [17, 50, 51].

In 2019, Croop and colleagues published results from a randomized, double-blind, placebo-controlled, Phase 3 study that assessed the safety, efficacy, and tolerability of a 75 mg dose of an ODT formulation of rimegepant in 1,351 patients who received treatment for a single migraine attack of moderate or severe intensity. Pain recurrence was defined as the “percentage of participants who were pain-free at 2 h[ours] but then later had some amount of pain” within 48 h of drug administration; rescue medications were permitted 2 h post-dose. Sustained pain freedom was defined as achieving pain freedom from 2 h to 24 or 48 h. Rescue medications included aspirin, ibuprofen, and acetaminophen (up to 1,000 mg per day), as well as naproxen (or any other nonsteroidal anti-inflammatory drug [NSAID]), antiemetics, or baclofen. Note that no second dose of rimegepant was allowed. Two hours after administration of the single dose of rimegepant ODT 75 mg, 21.2% (142 of 669) of patients reported pain freedom, compared with 10.9% (74 of 682) of those who received placebo. Sustained pain freedom from 2 to 48 h post-dose was reported in 13.5% (90 of 669) of patients in the rimegepant group, compared with 5.4% (37 of 682) of those in the placebo group. Additionally, 36.6% (52 of 142) of patients in the rimegepant group experienced recurrence from 2 to 48 h after the initial dose, compared with 50.0% (37 of 74) of patients in the placebo group (Table 1) [31]. This study utilized the most commonly reported definition of recurrence in the literature, which aligns with that published in the most recent IHS guidelines [2, 31]. This definition, however, may be misleading because use of rescue medications was not taken into consideration.

ACHIEVE I and ACHIEVE II were multicenter, randomized, double-blind, placebo-controlled, single-attack, Phase 3 trials that evaluated the safety, efficacy, and tolerability of ubrogepant in a conventional oral tablet formulation [32, 33]. In the two studies, an optional second dose of either ubrogepant or placebo, or rescue medication (the patient’s usual acute treatment for migraine), was permitted 2 to 48 h after the initial treatment for either a nonresponding or a recurrent migraine headache [51]. Rescue medications included acetaminophen, NSAIDs, opioids, antiemetics, and triptans [32, 33].

In 2019, Dodick and colleagues reported results from the ACHIEVE I study, in which a total of 1,436 patients with migraine were treated with an initial dose of placebo, ubrogepant 50 mg, or ubrogepant 100 mg. In the placebo group, the optional second dose consisted of two placebo tablets, whereas patients in the ubrogepant groups were rerandomized, so that those in the ubrogepant 50 mg group received either two placebo tablets or one 50 mg ubrogepant tablet and one placebo tablet, and those in the ubrogepant 100 mg group received either two placebo tablets or two 50 mg ubrogepant tablets. In the ubrogepant treatment arms, 19.2% (81 of 422) of patients in the 50 mg group and 21.2% (95 of 448) of those in the 100 mg group reported pain freedom at 2 h after the initial dose, compared with 11.8% (54 of 456) of those in the placebo group. Further, in the ubrogepant groups, 12.7% (53 of 418) of patients in the 50 mg arm and 15.4% (68 of 441) of those in the 100 mg arm reported sustained freedom from pain (i.e., pain freedom from 2 to 24 h after the initial dose without use of the optional second dose or rescue medication), compared with 8.6% (39 of 452) of those in the placebo group. In the pooled 50 mg and 100 mg ubrogepant groups, 38.6% (336 of 871) of patients took an optional second dose, with 20.0% receiving ubrogepant. Of patients who received ubrogepant 50 mg or ubrogepant 100 mg, 16.3% (69 of 423) of patients and 15.2% (68 of 448) of patients, respectively, used rescue medication for either a nonresponding or a recurrent migraine headache 2 to 48 h following an initial dose, compared with 28.7% (131 of 456) of those in the placebo group. It is unclear from the published data whether a second dose was taken because of an early recurrence or a lack of efficacy from a single dose (Table 1) [32].

In the 2019 ACHIEVE II study by Lipton and colleagues, a total of 1,465 patients with migraine received an initial dose of placebo, ubrogepant 25 mg, or ubrogepant 50 mg. Patients in the ubrogepant groups were randomized to receive placebo or to repeat the previous dose of ubrogepant for their optional second dose, whereas all patients in the placebo group received placebo as their optional second dose**.** In the ubrogepant groups, 20.7% (90 of 435) of patients in the 25 mg arm and 21.8% (101 of 464) of those in the 50 mg arm reported pain freedom at 2 h after the initial dose, compared with 14.3% (65 of 456) of patients in the placebo group. Sustained pain freedom was defined as pain freedom without the need for a second dose or rescue medication and with no occurrence thereafter of a moderate or severe headache 2 to 24 h after taking the initial dose. In the ubrogepant treatment arms, 12.7% (55 of 432) of patients in the 25 mg group and 14.4% (66 of 457) of those in the 50 mg group reported sustained pain freedom 2 to 24 h after the initial dose, compared with 8.2% (37 of 451) of patients in the placebo group. In the pooled 25 mg and 50 mg ubrogepant groups, 37.6% (338 of 899) of patients received an optional second dose of the study medication, compared with 42.8% (195 of 456) of those in the placebo group. Additionally, 20.5% of patients in the ubrogepant 25 mg group, 16.4% of patients in the ubrogepant 50 mg group, and 25.7% of those in the placebo group used rescue medication 2 to 24 h after the initial dose for either a nonresponding or a recurrent migraine headache (Table 1) [33].

In the ACHIEVE I and ACHIEVE II studies, use of an optional second dose of study medication, either ubrogepant or placebo, or rescue medication was indicative of recurrence of headache; however, these studies did not differentiate between the percentage of patients who took a second dose for a recurrent migraine headache and those who took it for a nonresponding headache [32, 33]. Therefore, at this time, it is difficult to determine the rate of recurrence in patients who were treated with ubrogepant.

### Lasmiditan

The serotonin 5-hydroxytryptamine (5-HT)_1F_ receptor agonist lasmiditan (REYVOW®, Eli Lilly USA, LLC, Indianapolis, IN) is the only FDA-approved ditan for the acute treatment of migraine [52–55]. The safety and efficacy of lasmiditan were evaluated in two large, single-attack, randomized, double-blind, placebo-controlled, Phase 3 trials, SAMURAI and SPARTAN. Sustained pain freedom was defined similarly for both studies, that is, the achievement of pain freedom at 2 h after the first dose and at the indicated assessment time, having not used any medications after the first dose [52, 53]. In 2018, Kuca and colleagues reported results from the SAMURAI study that evaluated the safety and efficacy of oral lasmiditan 100 mg and 200 mg in a total of 1,856 patients with migraine. The proportions of patients who reported sustained pain freedom at 24 and 48 h, respectively, were 18.6% (103 of 555) and 16.4% (91 of 555) of those in the lasmiditan 200 mg group, and 14.8% (83 of 562) and 14.9% (84 of 562) of those in the lasmiditan 100 mg group, compared with 7.6% (42 of 554) and 7.6% (42 of 554) of patients in the placebo group [52]. In 2019, Goadsby and colleagues reported results from the SPARTAN study, which was designed to confirm the safety and efficacy of three doses of oral lasmiditan, 50 mg, 100 mg, and 200 mg, in a total of 2,583 patients with migraine. The proportions of patients who reported sustained pain freedom at 24 and 48 h, respectively, were 22.7% (128 of 565) and 19.6% (111 of 565) of those in the lasmiditan 200 mg group, 17.9% (102 of 571) and 15.1% (86 of 571) of those in the lasmiditan 100 mg group, and 17.2% (103 of 598) and 14.9% (89 of 598) of those in the lasmiditan 50 mg group, compared with 13.4% (77 of 576) and 11.8% (68 of 576) of patients in the placebo group [53].

In 2019, Loo and colleagues conducted a post hoc analysis of data from the SAMURAI and SPARTAN trials that evaluated the safety and efficacy of a second dose of lasmiditan for rescue or recurrence. Whereas the individual studies reported only the percentages of patients taking a second dose regardless of whether it was for rescue or recurrence, this post hoc analysis clearly differentiated between the rescue and recurrence populations. Although patients in both populations took a second dose between 2 and 24 h after administration of the first dose, only those in the recurrence population were pain free at 2 h. The recurrence population was defined as “patients who achieved headache pain-free status at 2 h[ours] but then experienced recurrence of mild, moderate, or severe migraine pain and took a second dose of study drug up to 24 h[ours] from the first dose.” The proportions of patients who reported pain freedom in the ITT population (i.e., received a first dose of the study medication and experienced any post-dose headache severity or symptom assessments) at 2 h were 28% (169 of 598) in the lasmiditan 50 mg group, 30% (337 of 1,133) in the lasmiditan 100 mg group, and 35% (396 of 1120) in the lasmiditan 200 mg group, compared with 18% (206 of 1,130) in the placebo group. The proportions of patients who took a second dose for recurrence were 8% (13 of 169) in the lasmiditan 50 mg group, 10% (35 of 337) in the lasmiditan 100 mg group, and 7% (28 of 396) in the lasmiditan 200 mg group, compared with 10% (21 of 206) in the placebo group. The study also calculated the recurrence rate based on the definition used in earlier triptan studies. The recurrence population was defined as patients who achieved none or mild pain at 2 h, that is, 2-h pain relief after the first dose, and subsequently reported moderate or severe pain [3, 29]. Recurrence rates of 15.3% (53 of 346), 15.3% (107 of 698), and 14.1% (96 of 683) in the lasmiditan 50 mg, lasmiditan 100 mg, and lasmiditan 200 mg groups, respectively, compared with 17.4% (88 of 505) in the placebo group, were reported using this definition (Table 1) [29].

In 2019, Brandes and colleagues reported interim results from the prospective, randomized, open-label, Phase 3 GLADIATOR study. The investigators evaluated the long-term safety and efficacy of lasmiditan for the acute treatment of migraine in patients who had completed either the SAMURAI or the SPARTAN study. Patients were randomized in a 1:1 to ratio to lasmiditan 100 mg or lasmiditan 200 mg for up to 1 year. Patients who achieved headache pain-free status at 2 h but then experienced recurrence were permitted to take an optional second dose of lasmiditan up to 24 h after the first dose, provided they had not used any other migraine medication. Alternatively, patients could take their own medication for rescue or recurrence, but triptans, ergots, opioids, and barbiturates were not allowed within 24 h of lasmiditan administration. If lasmiditan was used for rescue or recurrence, responses were recorded in the patient’s e-diary up to 48 h after the second dose. The ITT population was defined at the treated migraine attack level (i.e., a migraine attack treated with ≥ 1 dose of lasmiditan in patients with any post-dose pain severity or symptom assessments for ≥ 1 migraine attack). Similarly, the mITT population was defined at the treated migraine attack level (i.e., all ITT migraine attacks treated within 4 h of onset). Pain freedom and relief analyses excluded treated migraine attacks with no pain severity rating at baseline or with a severity rating of ‘‘none’’. The proportions of migraine attacks with 2-h pain freedom in the ITT population were 26.9% (2,298 of 8,532) in the lasmiditan 100 mg group and 32.4% (2665 of 8232) in the lasmiditan 200 mg group, with 29.6% (4963 of 16,764) for all treated migraine attacks. The percentages of migraine attacks treated with medications other than lasmiditan before and after the 2-h assessment were 3.3% and 8.2%, respectively. In the ITT population, 6.1% (302 of 4963) of all treated migraine attacks that achieved pain freedom at 2 h were treated with a second dose of lasmiditan, whereas in the mITT population (*n* = 16,777), 17.1% of all treated migraine attacks that achieved pain freedom at 2 h had recurrence of pain up to 48 h post-dose (Table 1). Of all the treated migraine attacks that were pain free at 2 h in the mITT population, 10.1% and 8.7% in the lasmiditan 100 mg group and the lasmiditan 200 mg group, respectively, were treated with either a second dose of lasmiditan or with another medication [30].

The pooled analysis of the SAMURAI and SPARTAN studies allowed the use of an optional second dose of lasmiditan or rescue medication, but clearly defined which patients received a second dose for recurrence. This analysis first calculated the percentage of patients who took a second dose of study medication up to 24 h after the first dose who were initially headache pain free at 2 h, but then experienced recurrence of mild, moderate, or severe migraine pain. The study also calculated the recurrence rate based on those patients who became pain free or experienced mild pain at 2 h after the first dose, then subsequently reported moderate or severe pain within 24 h [3, 29], which clearly highlights how using two different calculations of recurrence provided varying results. The higher values obtained from the second definition may be due to including those patients who initially achieved mild pain at 2 h instead of those who were completely pain free at 2 h. In addition, the GLADIATOR study highlighted how recurrence rates can differ dramatically depending on which patient population is being assessed and which definition of recurrence is being used [30].

### Dihydroergotamine Mesylate (INP104)

A combination product (INP104) that delivers a nasal formulation of DHE mesylate using the Precision Olfactory Delivery (POD®) technology (Impel Pharmaceuticals, Seattle, WA) was FDA-approved in September 2021 (TRUDHESA®). Smith and colleagues reported results from the STOP 301 trial, which was a pivotal, interventional, open-label, Phase 3 study to assess the safety, tolerability, and exploratory efficacy of DHE mesylate administered with the POD technology to the upper nasal space over 24 or 52 weeks. A total of 354 patients comprised the full safety set (FSS; i.e., all patients who were enrolled in the study and received ≥ 1 dose of INP104). This set treated their migraine attacks with INP104 for up to 24 weeks, with a subset of 73 patients treating their migraine attacks for 52 weeks. Comparisons were made with data collected during the initial 4-week screening period, when patients were receiving their best usual care. For the first treated migraine, 38.0% (126 of 332) of patients self-reported pain freedom at 2 h with INP104, whereas 30.1% (92 of 306) of patients self-reported pain freedom at 2 h for their last migraine at baseline treated with best usual care. Recurrence was defined as the onset of a new headache prior to 24 or 48 h post-dose in patients who were pain free at 2 h post-dose. Recurrence at 24 h and 48 h post-dose was self-reported in 7.1% (9 of 126) of patients and 14.3% (18 of 126) of patients, respectively, who treated their first migraine attack with INP104 (Table 1) [34]. Sustained pain freedom was defined as a migraine with initial 2-h pain freedom with no recurrence and no other medication used between the time of INP104 usage and 24 or 48 h later. Sustained pain freedom was self-reported in 35.2% (117 of 332) of patients at 24 h and in 32.5% (108 of 332) of patients at 48 h for the first treated migraine attack. This study used a definition of recurrence (i.e., the onset of a new headache prior to 24 or 48 h post-dose in patients who were pain free at 2 h after a single dose) that aligned with the most commonly used definition in the literature and with the definition published in the most recent IHS guidelines [2, 34]. However, this definition differed in that it measured the onset of new headache, since 24- and 48-h pain measurements were not captured if a patient was pain free at 2 h in the STOP 301 study. Further, this definition did not consider rescue medication, although data on rescue medication use was collected. A limitation of this study is the open-label design, which precluded a placebo comparison; however, data from STOP 301 demonstrated that the first migraine attack treated with INP104 was associated with low rates of recurrence through 24 and 48 h post-dose [34].

## Conclusion

In this review, rates of recurrence with acute therapies for migraine were presented based on how the study defined recurrence. The substantial numerical differences in recurrence rates reported in the literature and the lack of alignment in the definition of recurrence between clinical trials render it difficult to accurately compare data among the different clinical studies. It is important to note that comparing recurrence rates with different agents in isolation may be misleading and does not necessarily represent the long-lasting effect of any particular drug by itself. Further, differences in study type (e.g., open-label vs placebo-controlled) can contribute to this misrepresentation. In general, understanding recurrence in migraine requires evaluation of the definition of recurrence. Defining recurrence to include both the return of pain and utilization of rescue medication but limiting the definition to the population of patients who experience initial pain freedom may provide a more complete description of meaningful persistence of migraine relief. A standardized definition is required to help physicians compare the rates of recurrence among different acute treatments for migraine, which may ultimately lead to improved quality of life among patients with migraine. The sustained pain-free definition set by the IHS is a better outcome measure of prolonged efficacy than recurrence rate for acute medication trials.

## Data Availability

Appropriately anonymized data reported for the STOP 301 study are available by request from Impel Pharmaceuticals—the company sponsoring the clinical development of INP104 for the acute treatment of migraine. The MAP0004 data are available by request from Stewart J. Tepper.

## Abbreviations

5-HT: 5-Hydroxytryptamine
DHE: Dihydroergotamine
FSS: Full safety set
IHS: International Headache Society
ITT: Intent-to-treat
mITT: Modified intent-to-treat
ODT: Orally disintegrating tablet
